# A Novel Prothrombotic Pathway in Systemic Sclerosis Patients: Possible Role of Bisphosphonate-Activated γδ T Cells

**DOI:** 10.3389/fimmu.2014.00414

**Published:** 2014-09-08

**Authors:** Victoria Marcu-Malina, Alexandra Balbir-Gurman, Rima Dardik, Yolanda Braun-Moscovici, Michael J. Segel, Ilan Bank

**Affiliations:** ^1^Laboratory of Immunoregulation, Sheba Medical Center, Ramat Gan, Israel; ^2^B Shine Rheumatology Unit, Rambam Health Care Campus, Rambam Medical Center, Haifa, Israel; ^3^Institutes of Thrombosis and Hemostasis, Sheba Medical Center, Ramat Gan, Israel; ^4^Institute of Pulmonary Diseases, Sheba Medical Center, Ramat Gan, Israel; ^5^Department of Medicine F, Sheba Medical Center, Ramat Gan, Israel; ^6^Department of Medicine, Sackler School of Medicine, Tel Aviv University, Tel Aviv, Israel

**Keywords:** T cells, γδ T cell, scleroderma, tissue factor, thrombosis, aminobisphosphonate, Vγ9δ2 T cells

## Abstract

**Objectives:** Infusions of aminobisphonates (ABP) activate Vγ9δ2T cells *in vivo* and induce an acute inflammatory response in 30% of patients treated for osteoporosis. Following the observation of digital thrombosis in a systemic sclerosis (SSc) patient after treatment with an intravenous ABP, zoledronate (Zol), we evaluated whether patient and control peripheral blood (PB) mononuclear cell (MC, PBMC) acquire a prothrombotic phenotype in response to Zol.

**Results:** Vγ9δ2T cells of both patients and healthy donors (HD) upregulated the CD69 activation antigen and secreted tumor necrosis factor (TNF)α in response to Zol *in vitro*. In addition, exposure to either Zol or lipopolysaccharide (LPS), or to both additively, induced expression of the highly procoagulant, tissue factor (TF)-1 on CD14+ monocytes. Importantly, only Zol-induced TF-1 was blocked by a monoclonal antibody to TNFα. Interestingly, we found that SSc, but not HD, Vδ1+ T cells were concurrently activated by Zol to produce interleukin (IL)-4. Addition of plasma from the blood of the SSc patient who developed critical digital ischemia after infusion of Zol, but neither plasma from a second patient with no adverse clinical response to Zol infusion nor of a HD, strongly enhanced Zol-induced monocyte TF-1, which could still be blocked by anti-TNFα.

**Conclusion:** Aminobisphonates induced secretion of TNFα by Vγ9δ2+ T cells may lead to TNFα-dependent induction of procoagulant TF-1 induction on monocytes. In certain clinical settings, e.g., SSc, TF-1+ monocytes could play a role in triggering clinically relevant thrombosis.

## Introduction

γδ T cells are a subset of T cells combining innate and adaptive functions (1). In Caucasians, 50% of the circulating γδ T cells express the γ9 and δ2 genes in the Variable (V) region of the γδ T cell receptor (TCR) Vγ9δ2 T cells (2). Vγ9δ2 TCR recognize metabolites produced in the classical (isopentenyl pyrophosphate, IPP) and alternative [(E)-4-hydroxy-3-methyl-but-2-enyl pyrophosphate (HMBPP)] mevalonate metabolic pathways. These antigens are presented for TCR-mediated recognition by CD277, a ubiquitously expressed cell surface membrane antigen presenting molecule (APM) (3). Together with co-stimulatory signals delivered by antigen presenting cells (APC), Vγ9δ2 TCR–CD277/IPP cognitive interactions activate the Vγ9δ2 T cells to secrete cytokines and exert cytotoxic effects. A second major subset of γδ T cells expresses the Vδ1 gene in the TCR structure, among which a major portion recognize phospholipid antigens (e.g., sulfatide) presented by CD1 family molecules (4).

Vδ1 γδ T cells have been shown to expand oligoclonally in the PB of certain systemic sclerosis (SSc) patients, infiltrate the skin in early phases of the disease, and may secrete factors enhancing collagen production (5). Vγ9δ2 T cells are also functional in SSc patients, since their circulating Vγ9δ2 T cells secrete tumor necrosis factor (TNF)α and IFNγ and induce fibroblast apoptosis in the presence of exogenously added IPP (6). However, the immunopathogenic significance of these cells concerning the clinical manifestations in SSc patients remains largely unknown.

In this regard, interactions of γδ T cells with bisphosphonate-activated CD14+ monocytes may play a critical role. Bisphosphonates block farnesylpyrophosphate synthase (FPPS) downstream of IPP in the mevalonate pathway in circulating CD14+ monocytes, increasing intracellular IPP, which is presented to circulating Vγ9δ2 T cells leading to their activation (7, 8). As a consequence, these cells produce TNFα and IFNγ, the central mediators of the acute phase response (APR) following infusion of zoledronate (Zol) to patients (8). Accordingly, upon administration of a bisphosphonate drug for osteoporosis or to decrease bone metastasis in cancer, an APR characterized by fever, chills, and arthralgia occurs in up to 30% of patients (8). Zol also activates dendritic cells and natural killer (NK) cells at least in part dependent upon Vγ9δ2 T cell activation (9, 10). CD86 and other stimulatory molecules, which enhance activation of other T cell subsets are also upregulated by Zol on dendritic cells (11). Recently, Zol was also shown to stimulate B cells directly (12). Our recent observation of the rapid onset of gangrene of fingers and toes in a patient with SSc after Zol administration prompted the experiments in this study that were designed to evaluate how bisphosphonate-activated γδ T cells could play a pathogenic role in SSc.

## Materials and Methods

### Patients

The study was approved by the Institutional Review Board (Helsinki Committee) of the Sheba Medical Center, Ramat Gan, Israel. All patients participating in the study were seen in the Rheumatology and Pulmonary clinics at Sheba or Rambam medical centers. Patients fulfilled the criteria of the American College of Rheumatology for systemic sclerosis (SSc), also named herein scleroderma (SCL) (13). Controls included six healthy donors (HD), two patients with idiopathic pulmonary fibrosis (IPF), and one patient with polymyalgia rheumatica (PMR).

### Isolation of PBMC and characterization of cell subsets

PBMC were isolated by Lymphoprep (AXIS-SHIELD, Oslo, Norway) density centrifugation and cultured in growth medium as previously described (14). Cells were stained with fluorochrome conjugated monoclonal antibodies (mAb) specific to CD4, CD3, CD142, CD14, CD69, or isotype control (BD Biosciences), or to human Vγ9, Vδ2 (Immunotech), and Vδ1 (Endogen, Pierce) and analyzed by flow cytometry (Calibur, Beckton Dickinson, CA, USA).

### Tissue factor induction and inhibition

PBMC were incubated and stimulated either with 2 μM of Zol (Novartis) overnight (ON) or with 50 ng/ml of *E. coli* 0111:B4 lipopolysaccharide (LPS) (InvivoGen) for 3 h. For double LPS + Zol stimulation, cells were cultured with Zol ON then LPS was added for an additional 3 h. Cells were harvested, washed, and stained with CD14 and CD142 specific antibodies. For inhibition experiments, PBMC from HD were cultured in medium with increasing dilutions of either anti-TNFα antibody (Infliximab, Janssen Biologics) or control IgG mix (Gammaplex, Bio Products Laboratory, Herts, UK) prior to stimulation with Zol or/and LPS.

### Cytokine detection

Intracellular cytokine detection was performed as previously reported (14). IFNγ detection in supernatants was done using the ELISA max Deluxe Sets (Biolegend, CA, USA) according to the manufacturer’s instructions.

## Results

### SSc-disease specific response of Vδ1+ T cells to Zol

**Figure 1:**
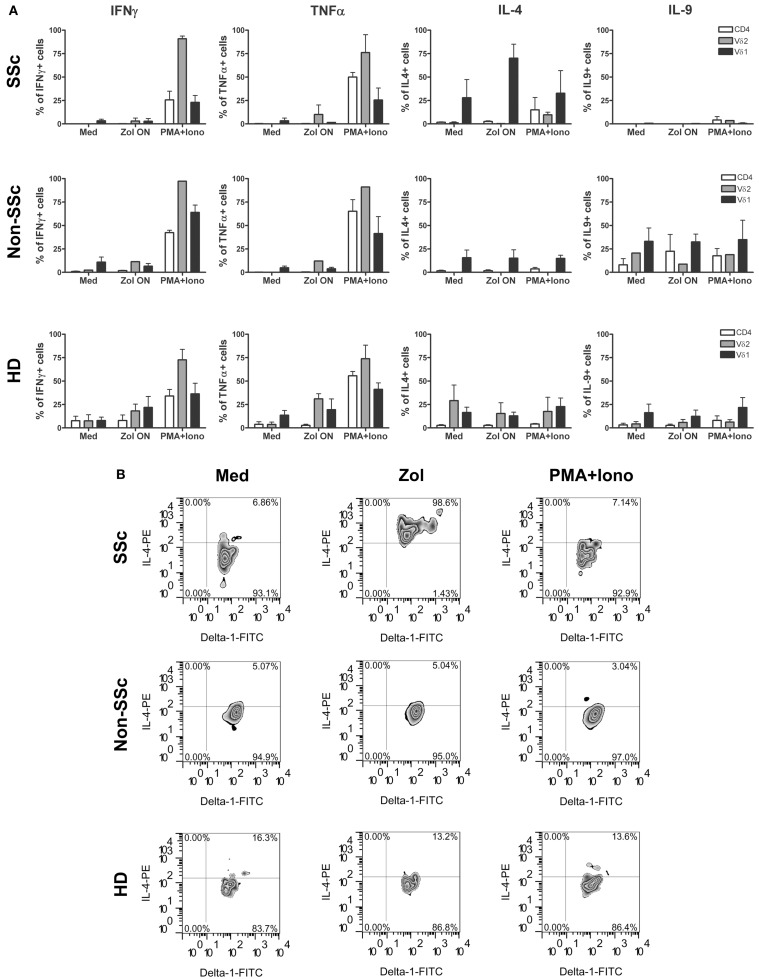
**Cytokine expression pattern in T cell subsets in systemic sclerosis (scleroderma, SSc), control patients, and healthy donors is shown**. Bars represent percent cells in the indicated T cell subsets ±1 SEM after activation of PBMC from healthy donors (HD) (*n* = 5) or scleroderma (SSc) patients (*n* = 3) or non-scleroderma (SSc) control patients (*n* = 3) following stimulation with Zol, PMA, and ionomycin or medium (Med) **(A)**. **(B)** Representative plots show IL-4 secretion by Vδ1+ T cells from RP-2, a control patient, or a HD in response to Zol, PMA + Ionomycin, or medium.

Activation of γδ T cells in SSc patients was compared to that of healthy individuals and patients with other chronic inflammatory diseases quantitating percentage of cells producing a panel of relevant intracellular cytokines. Thus, PBMC were incubated ON with Zol and secretion of cytokines was measured by intracellular staining of PB T cell subsets. Percentage of cells among the CD4+, Vγ9δ2+, and Vδ1+ T cell subsets in PBMC of 3 SSc patients (RP0-2), 3 non-SSc patients with IPF or PMR (Pt 1–3), and 6 HD that were induced to secrete IFNγ, TNFα, IL-4, or IL-9 after 4.5 h of incubation in medium alone, or with Zol or PMA (20 ng/ml) and ionomycin (0.8 μM) (P/I) are shown in Figure 1. A significantly higher percentage of IL-4 producing cells was observed among SSc patient’s Vδ1+ cells exposed to Zol compared to either non-SSc patients (*p* < 0.03, Student T test) or HD (*p* < 0.003, Student T test). In contrast, there was no significant difference in the mean percentage of cells secreting any of the other cytokines in SSc compared to HD in the remaining T cell subsets. SSc patient IFNγ production was, however, significantly lower than in the non-SSc patients among Zol-activated CD4+ T cells, and P/I-activated Vδ1+ T cells. Among SSc Vδ1+ T cells, the percentage of Zol-activated IL-9 producers were also significantly lower than in non-SSc patients (*p* < 0.05). These results point to a unique pattern of production of cytokines of SSc Vδ1+ γδ T cells in response to Zol, relative to HD and patients with other chronic inflammatory and fibrotic diseases, characterized by increased production of IL-4 (relative to both HD and disease controls), and decreased production of IL-9 relative to the disease controls.

### Activation of patient RP2 Vγ9δ2+ T cells by Zol

**Figure 2:**
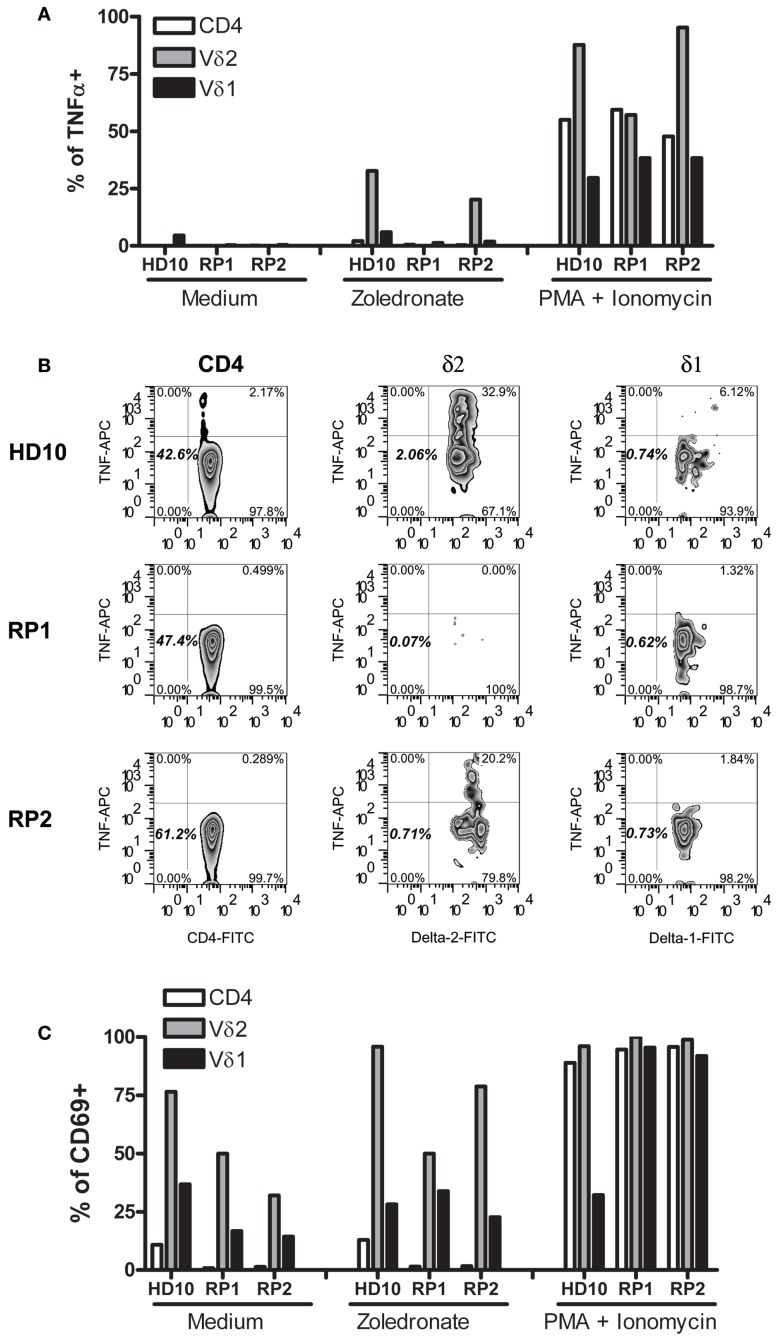
**Vγ9δ2 T cells of patient RP2 produced TNFα in response to zoledronate**. Bars represent percent cells of HD10, and of SSc patients RP2 and RP1, within the indicated T cells subsets expressing intracellular TNFα **(A)** or surface CD69 **(C)** after stimulation with medium, Zol or PMA, and ionomycin. **(B)** Representative FACS plots of TNFα staining in the corresponding T cells from patients indicated on the left after Zol stimulation. Numbers indicate percentages of the particular cell population in the respective rectangle.

During the course of these studies, only one of our SSc patients (RP2) developed an unusually dramatic APR after receiving intravenous Zol. Since TNFα and IFNγ produced by Vγ9δ2 γδ T cells are thought to be the mediators of the APR and IFNγ was weakly produced in SSc patients in response to Zol (Figure 1), we examined in further detail how Zol had affected TNFα production by this patient’s (RP2) PB T cell subsets. As expected, CD4+, Vγ9δ2 as well as Vδ1+ T cells in PBMC of RP2, RP1, and a healthy blood donor all increased their intracellular TNFα in response to P/I, an activating stimulus for T cells that bypasses signals dependent upon cognitive TCR-antigen interactions. Zol potently induced RP2 Vγ9δ2 T cells (but not CD4+ or Vδ1+ T cells) to produce TNFα, similar to its effect on a HD PBMC (Figure 2) whereas those of RP1 an SSc patient who had received Zol but no clinical APR did not secrete TNFα in response to Zol application *in vitro*. Production of TNFα was linked to Zol-dependent activation of Vγ9δ2+ γδ T cells. Thus, a markedly increased expression of CD69 on the surface of Vγ9δ2 cells (but not Vδ1+ cells or CD4+ T cells) was concomitantly noted in the presence of Zol on RP2 and HD but not on RP1 Vγ9δ2+ T cells. In contrast, P/I stimulation increased CD69 expression in all T cell subsets in all individuals tested (except in HD Vδ1+ cells).

### Induction of TNFα-dependent tissue factor on CD14+ monocytes by Zol

**Figure 3:**
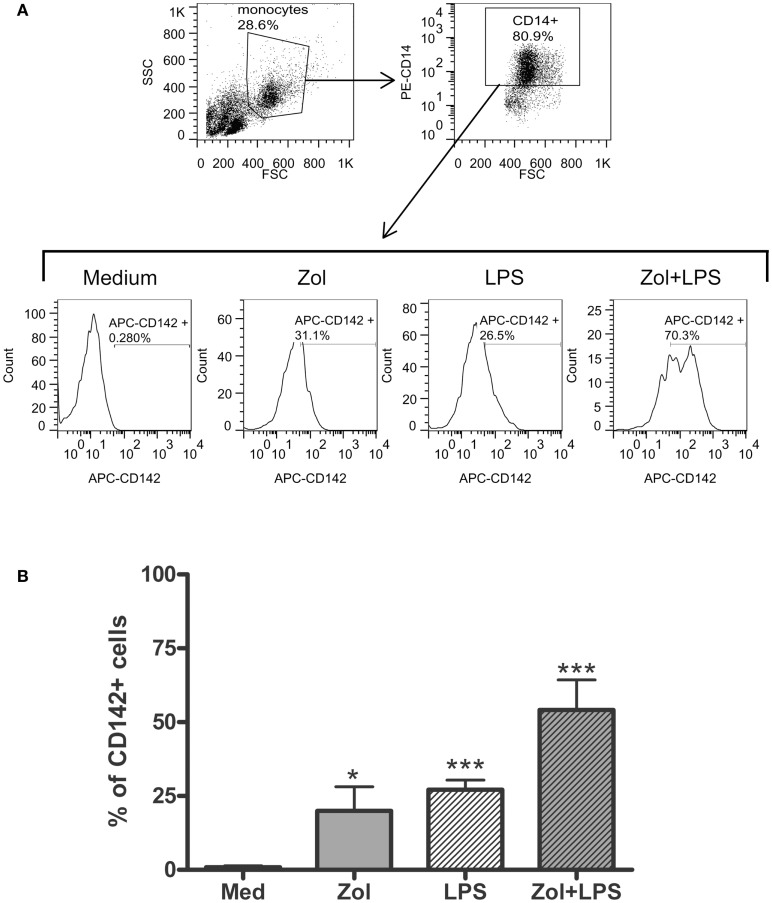
**Zoledronate induces tissue factor-1 (TF-1) surface expression on CD14^+^ monocytes**. **(A)** Histograms in the second panels depict TF-1 expression as percent CD142+ among CD14+ monocytes in PBMC cultured in medium or after stimulation with Zol, LPS, or with both, as indicated (see Materials and Methods). The panel above shows forward (FSC) and side scatter (SSC) dot plots of the PBMC gating used to identify monocytes and expression of CD14 in the gated monocyte subset. **(B)** Summary of five independent experiments showing %CD142+ monocytes in cultures of HD PBMC as above. *p < 0.05, ***p < 0.0001.

The occurrence of digital ischemia during the APR in RP2 prompted us to examine whether TNFα produced by Vγ9δ2+ cells in response to Zol is sufficient to induce tissue factor (TF)-1, a potent procoagulant factor, on the cell surface membrane of monocytes present in PBMC (15). We utilized HD PBMC to address this issue, because we could obtain only two PB samples from patient RP2 and the patient’s response to Zol with respect to TNFα secretion was similar to that of HD (Figure 2). Thus, HD PBMC were incubated ON in medium alone or medium containing Zol. Subsequently, LPS, a known inducer of TF-1 on monocytes, was added to the medium for an additional 3 h. The expression of the CD142 antigen, which identifies TF-1 on the cell surface, was assessed by FACS analysis after gating on CD14+ monocytes. A marked and significant upregulation of CD142 on HD-derived monocytic CD14+ cells cultured with either Zol alone ON or after the brief 3 h LPS stimulation was observed (Figure 3). There was no upregulation of CD14 in these experiments, indicating that upregulation of CD142 was not due to non-specific elevation of surface membrane molecules on monocytes (data not shown). Furthermore, an additive effect on CD142 levels of expression was noted in PBMC cultured in the presence of both reagents (Figure 3).

**Figure 4:**
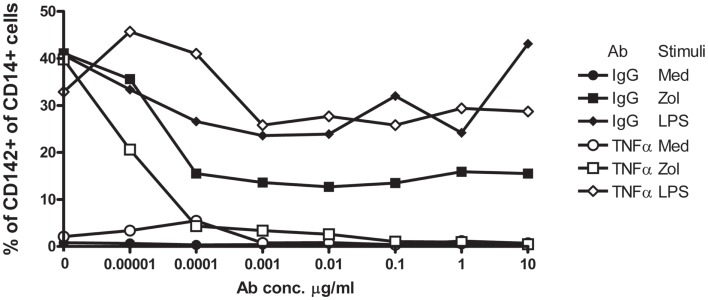
**Upregulation of TF-1 in response to Zol is TNFα-dependent**. PBMC from a healthy donor were cultured in medium with graded dilutions (indicated on the *x* axis) of either anti-TNFα mAb or control IgG. Cells were stimulated with Zol, with LPS or not stimulated (medium), and TF-1 was measured by flow cytometry as described in Figure 3. Results are representative of three experiments.

We next performed similar experiments and included infliximab, a TNFα neutralizing humanized mAb, or similar concentrations of non-specific human IgG, in parallel cultures. Infliximab abolished, in a dose-dependent manner, induction of TF-1 on the surface membrane of CD14+ monocytes in response to Zol but not to LPS (Figure 4). Although control IgG also slightly reduced TF-1 expression induced by Zol, inhibition was incomplete even at high concentrations of IgG. Together, these results indicate that Zol, when added to PBMC, induces TNFα secretion by the Vγ9δ2+ T cells in the PBMC and TNFα-dependent expression of TF-1 on CD14+ monocytes.

### Effect of patient plasma on TF-1 induction

**Figure 5:**
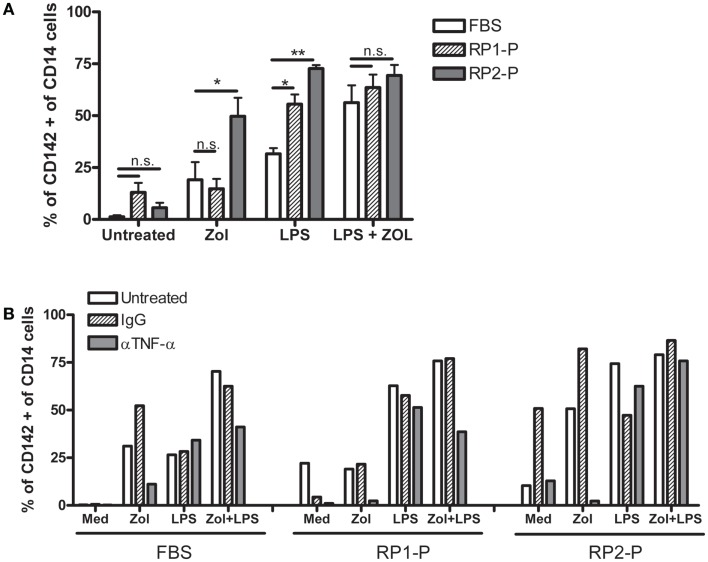
**RP2-plasma increases TNFα-dependent upregulation of TF-1 in response to LPS and Zol**. **(A)** Bars represent percent ± 1 SEM of CD14+ cells expressing TF-1 (CD142) in PBMC cultures from three HDs, cultured with the indicated stimuli on the *X* axis, in the presence of medium containing 10% FBS or RP1- or RP2-plasma (RP1-P or RP2-P). **(B)** Shows the same experiment done in the presence of 10 μg/ml anti-TNFα antibody or control IgG as indicated.

Zoledronate has not been previously reported to induce digital necrosis despite its widespread use in patients. Furthermore, in our experience, which included four SSc patients, only RP2 developed an APR and critical digital ischemia, in which TF-1 may have contributed. It was thus of interest to determine the role of RP2 patient specific factors in the development of the unusual response to Zol. We found that addition of RP2-plasma (RP2-P) markedly increased TF-1 induction on CD14+ monocytes in response to Zol as well as LPS, whereas control plasma (RP1-P) from SSc patient RP1 who had received Zol with no toxic effect did not enhance TF-1 induction (Figure 5). Importantly, addition of anti-TNFα mAb but not of non-specific IgG still completely abolished TF-1 upregulation in response to Zol even in the presence of RP2-P. Anti-TNFα mAb did not reduce TF-1 upregulation in response to LPS or the combination of LPS and Zol in the presence of RP2-P. These results suggest that the patient’s plasma specifically contained factor/s that enhance Zol-induced Vγ9δ2+ produced TNFα-dependent TF-1 expression on monocytes *in vitro*, suggesting that a similar effect may have taken place following IV infusion on her circulating monocytes.

## Discussion

This paper shows, for the first time, an SSc specific effect of Zol, i.e., the enhancement of IL-4 secretion by PB Vδ1+ T cells, coupled with diminished ability of these cells to secrete IL-9 relative to two patients with another form of fibrosing disease, IPF. Furthermore, Zol induced secretion of TNFα by Vγ9δ2+ T cells from some SSc patients similar to healthy individuals, which in turn induced TF-1 on CD14+ monocytes. Finally, plasma of an SSc patient who suffered a clinically detrimental inflammatory response strongly augmented this TNFα-dependent TF-1 expression on monocytes. These data suggest that activation of both major γδ T cell subsets – Vδ1+ and Vγ9δ2+ by Zol could activate pathogenic mechanisms, e.g., fibrosis (via IL-4) and thrombosis (via TF-1) – relevant to clinical manifestations in SSc patients.

The bisphosphonate-induced APR in patients receiving intravenous therapy for osteoporosis or cancer differs from a typical APR. Thus, CD14+ monocytes and macrophages are the primary cytokine producing cells in the latter, whereas bisphosphonates induce rapid and copious production of TNFα, IFNγ, and IL-6 primarily by PB Vγ9δ2 T cells (16). Activation of the Vγ9δ2+ T cells triggered by upregulation of IPP in monocytes in turn enhances CD14, CD40, CD80, and HLA-DR on circulating monocytes (8). Zol also enhances TNF-related apoptosis-inducing ligand (TRAIL) in γδ T and NK cells, and release of high mobility group box 1 (HMGB1) from γδ T cells and monocytes (17). Furthermore, soluble factors released by activated Vδ2/monocytes co-cultures induce granulocyte migration and activation (18). Activated Vγ9δ2T cells also trigger granulocyte functions via MCP-2 release during bacterial infection (9).

The novel finding shown here, i.e., induction of TF-1 on monocytes, in a manner dependent upon TNFα produced by Vγ9δ2+ T cells stimulated with Zol, adds an additional dimension to the role of monocyte–Vγ9δ2 interactions, that may play a critical role in clinical medicine, since TF-1 expressing monocytes play an important role in thrombotic diseases (19). For example, patients with cardio- and cerebro-vascular disease have increased TF-1 expression on circulating monocytes and TF-1-positive monocyte-derived circulating microparticles in the blood (20, 21). Furthermore, circulating monocyte-derived microparticles expressing TF-1 are associated with acute recurrent deep venous thrombosis (22). In experimental hypercholesterolemic mice, the associated prothrombotic state is caused by oxidized low density lipoprotein engagement of a toll-like receptor (TLR)4/TLR6 complex, leading to induction of TF-1 in monocytes (23). In addition, in rats, monocytes in blood vessels of kidneys undergoing acute rejection express high levels of TF-1 (24). These data suggest that Zol infusion, which achieves concentrations of zoledronate in the plasma similar to those we have used in our experiments, may lead to induction of TF-1 on circulating monocytes resulting in a prothrombotic state, which may have contributed to development of ischemic digits in SSc RP2 patient described here (25). Our data, furthermore, show that Zol-induced TF-1 on monocytes is at least partly dependent upon Vγ9δ2 cell produced TNFα (Figures 1 and 5). In contrast, a combination of mAb to TNFα and IL-1β, but neither alone, was required to inhibit high molecular weight kininogen induced monocyte TF-1 (26). TNFα may also upregulate monocyte TF-1 activity indirectly via its effects on endothelial cells (27, 28).

Although Zol induced increase of TF-1 on monocytes, overt thrombosis in the absence of additional factors is rare. Nevertheless, inclusion of zoledronic acid in the treatment protocols for multiple myeloma (MM) and breast cancer significantly increases venous thrombosis (29–32). In this regard, Vγ9δ2+ T cells may be playing a role, since in both MM and breast cancer patients, these cells are known to become activated by Zol (33–36). These data suggest that Zol-induced thrombosis is dependent upon disease and/or patient specific factors, which may include Zol responsive Vγ9δ2+ T cells in the PB and additional factors.

Our data suggest that SSc may constitute a risk for severe prothrombotic γδ T cell-mediated Zol-induced reactions. In this regard, the already diseased endothelium in SSc may play a role, since activated γδ T cells of SSc patients, in particular, adhere to and damage endothelial cells, creating a substrate for enhancing thrombosis (37). The current data suggest that at least two additional factors could play a role in Zol-induced thrombosis in SSc patients. First, SSc patient’s plasma may contain factors that enhance TF-1 expression in response to Zol [Figure 5 and Ref. (16)]. Indeed, SSc plasma has been shown previously to contain increased levels of circulating TNFα, platelet microparticles, and soluble CD40 ligand, which could collaborate in the induction of TF-1 (38). That TF-1 induction in the setting of exposure to Zol can be blocked by an anti-TNFα antibody even in the presence of enhancement by patient’s plasma (Figure 5) suggests that TNFα released by Zol-activated Vγ9δ2+ T cells plays a major role in induction of TF-1 and that anti-TNFα mAb could be used to prevent thrombosis in high risk SSc patients treated with Zol. In addition, our study is the first, to our knowledge, to describe a disease-specific IL-4 response of SSc patients Vδ1+ T cells to Zol (Figure 1). A bias toward IL-4 secretion by SSc patients CD4+ TCR αβ T cells in response to non-specific stimulation has already been observed (39). Moreover, Vδ1+ γδ T cells often predominate in the context of a Th2-biased environment, e.g., in the broncho-alveolar lavage fluid obtained from allergic individuals (40, 41). In addition, the majority of phosphatidyl-ethanolamine CD1d-restricted γδ T clones in allergic individuals are Vδ1+ and secrete high levels of IL-4 (42). We hypothesize that Zol stimulation may upregulate co-stimulatory monocytes CD40, CD80, and CD1d molecules, which in turn, enhance IL-4 secretion by CD1d-restricted lipid antigen responsive Vδ1+ T cells in the PB of SSc patients (43, 44). The combined effects of the TNFα secreted by Vγ9δ2 cells, together with the IL-4 produced by Vδ1+ T cells increases vascular cell adhesion molecule 1 (VCAM-1) expression on endothelial cells in digital arteries and VCAM-1-mediated adhesion of TF-1+ monocytes to the endothelium could then activate local thrombosis and gangrene (45).

In summary, the hitherto described disastrous consequences of a seemingly innocuous and highly utilized drug, Zol, in an SSc patient, which prompted these investigations, led to the discovery of a novel prothrombotic pathway involving Vγ9δ2 γδ T cells and CD14+ monocytes and a disease-specific activation of IL-4 producing Vδ1 γδ T cells. Future studies into this pathway may lead to new insights into the immunopathogenic mechanisms of thrombotic diathesis in immune-mediated, infectious, and malignant diseases, in which γδ T cells play a role.

## Conflict of Interest Statement

The authors declare that the research was conducted in the absence of any commercial or financial relationships that could be construed as a potential conflict of interest.
